# Risky Business: Do Native Rodents Use Habitat and Odor Cues to Manage Predation Risk in Australian Deserts?

**DOI:** 10.1371/journal.pone.0090566

**Published:** 2014-02-28

**Authors:** Emma E. Spencer, Mathew S. Crowther, Christopher R. Dickman

**Affiliations:** Desert Ecology Research Group, School of Biological Sciences, The University of Sydney, Sydney, New South Wales, Australia; Bangor University, United Kingdom

## Abstract

In open, arid environments with limited shelter there may be strong selection on small prey species to develop behaviors that facilitate predator avoidance. Here, we predicted that rodents should avoid predator odor and open habitats to reduce their probability of encounter with potential predators, and tested our predictions using a native Australian desert rodent, the spinifex hopping-mouse (*Notomys alexis*). We tested the foraging and movement responses of *N. alexis* to non-native predator (fox and cat) odor, in sheltered and open macro- and microhabitats. Rodents did not respond to predator odor, perhaps reflecting the inconsistent selection pressure that is imposed on prey species in the desert environment due to the transience of predator-presence. However, they foraged primarily in the open and moved preferentially across open sand. The results suggest that *N. alexis* relies on escape rather than avoidance behavior when managing predation risk, with its bipedal movement probably allowing it to exploit open environments most effectively.

## Introduction

Olfactory recognition of predators is especially widespread in mammalian predator-prey systems (for a review see: [1]). Typical responses by prey to predator odors include reduced foraging or general activity (e.g. [2]) and, in small mammals, lower trapping success at sites scented with predator odors [3]. Predator scent may indicate sites where there is an elevated risk of encountering a predator, and thus animals that use olfaction to detect and avoid these riskier habitats are likely to be at a selective advantage compared to those that do not [4], [5].

Predation plays a key role in shaping the dynamics and composition of native biota in many local and regional areas of Australia [6]–[8]. Of particular consequence is predation from the introduced European red fox (*Vulpes vulpes*) and the feral cat (*Felis catus*), which have become established over the past 150 years [9]. The large impacts of these predators could be expected to drive native prey to develop strategies to manage predation risk; however, studies on the use of odor cues by mammalian prey species in Australia have yielded contradictory results. Some native species avoid the odors of all predators [10]–[12], while others appear to respond only to the odors of native predators [13]. In some cases, native mammals show no evident avoidance of the odors of native or introduced predators [14], [15].

The potential use of predator odor by prey species might be readily explored using predator-prey systems in arid regions. High predator densities in deserts [16], [17] and the prevalence of camouflage coloration among desert animals [17] indicate that predation may be an important selective agent in arid landscapes. The low productivity of arid environments may also increase the pressure that predators can impose on prey species, as detection of prey is typically easier for visually-hunting predators where vegetation cover is sparse [18]. In Australian arid environments dramatic resource pulses trigger population irruptions of primary consumers, which constitute the prey base for larger predators [19], [20]. Following these population irruptions, predation risk may be considerable, and predators then are expected to exert strong selective pressure on prey species [19].

Although productivity is low, arid environments are often characterized by strongly heterogeneous habitat cover [21]. Many prey species perceive open habitats as risky and hence avoid them to reduce their chances of encounter and detection by predators (e.g. [22], [23]). Australian arid environments also experience frequent fires and long droughts. These events can accentuate the already-pronounced spatial variation in habitat cover by increasing the extent of open habitat [24] and, in turn, increase the need for prey individuals to respond flexibly to differing levels of predation risk. After patchy, low intensity fires rodents may forage similarly in burnt and unburnt habitat, but after intense broadscale fires that leave little above-ground cover, they may move only in small residual areas where shelter is available [25], [26]. It is therefore likely that, in addition to predator odor, prey animals in such systems also use structural cues to manage predation risk at both the macrohabitat (broad vegetation type) and microhabitat (habitat component) scales.

In the Simpson Desert, central Australia, the spinifex hopping-mouse (*Notomys alexis*) is depredated frequently by cats and foxes (e.g. [27], [28]) and hence, during periods of elevated predator activity, could be expected to be under strong selection pressure to reduce predation risk. We aimed to examine the foraging and movement responses of *N. alexis* to cat and fox odor as cues for predation risk. *Notomys alexis* tends to forage near cover, suggesting that animals may perceive open areas as relatively risky [29]. Thus, we also aimed to quantify the responses of this species to habitat cover, measured at both micro- and macrohabitat scales. The macrohabitat comparison was attained by using burnt and unburnt sites to represent areas with extensive (unburnt) and sparse (burnt) vegetation cover, whereas microhabitats comprised sites under vegetation or in open sand.

We predicted that *N. alexis* would:

1. forage less actively in areas with predator odors than in areas where no odors were added,
2. forage less actively in open microhabitats and macrohabitats than in microhabitats and macrohabitats that provide shelter,
3. forage less actively in open areas with predator odors than in areas with either no predator odors, or sheltered habitats, or both, and
4. after foraging, select sheltered movement paths more strongly in open areas with predator odors than in areas with either no predator odors, or with sheltered habitats.

## Methods

### Ethics Statement

This study was conducted on leasehold land with permission of the leaseholder, Bush Heritage Australia, and in accordance with *The Australian Code of Practice for the Care and Use of Animals for Scientific Purposes (1997)*. Research was approved by the University of Sydney Animal Ethics Committee (approval # L04/4-2009/3/5020).

### Study Species

*Notomys alexis* is a terrestrial native rodent (Muridae) found across most of arid Australia in hummock and tussock grasslands, sand dunes, arid eucalypt woodlands and acacia scrublands [30]. The species is nocturnal and omnivorous, eating seeds, invertebrates and green plant material [31]. The adult body mass of *N. alexis* is approximately 30 g. During drought, densities can be <0.1 animals per hectare, but after heavy rain, densities expand to >25 animals per hectare [30].

### Study Sites

We conducted experiments at three sites on Ethabuka Reserve in the Simpson Desert, central Australia. Site 1 (23°52′S, 138°28′E), site 2 (23°45′S, 138°28′E) and site 3 (23°40′S, 138°26′E) were spaced >10 km apart and all included burnt and unburnt habitats separated by >500 m. The sites were characterized by long, parallel sand dunes that ran NNW–SSE, about 0.6–1 km apart and 8–10 m high [32]. Vegetation was dominated by spinifex grass (*Triodia basedowii*: Poaceae), which occurred extensively in the interdunal areas (swales). Gidgee (*Acacia georginae:* Mimosaceae) was the most common tree species, occurring on patches of clay soil in the swales. The dune crests supported several species of ephemerals and shrubs including *Crotalaria* spp. (Fabaceae), *Calotis erinacea* (Asteraceae), *Tephrosia rosea* (Fabaceae), *Goodenia cycloptera* (Goodeniaceae) and *Grevillea stenobotrya* (Proteaceae) [33], and typically provided more open sand than the swales. About 25% of the reserve burnt in 2011 in both low and high intensity fires [34]. Prior to this study, on-site rainfall was low, with a total of 34 mm of rain falling from January–November 2012. However, from January 2010–April 2011, 950 mm of rain was recorded (rainfall data taken from a pre-existing Environdata weather station located at site 1).

### Field Sampling and Experimental Manipulations

Two experiments were carried out in sequence, with the results of Experiment 1 (completed in September 2012) used to inform the procedures used in Experiment 2 (completed in November 2012). Strong moonlight significantly improves predator hunting success and foraging prey may reduce their activity under these conditions [35]. Thus, as our experiments were conducted at night, to minimize this potential confounding factor, we conducted our tests under a new moon and completed them early in its first quarter. Before beginning Experiment 1, foraging stations set up in pilot trials showed that rodent activity was virtually absent on the dune sides and swales, so dune crests were selected as the focus for both experiments. In Experiment 1, one burnt and one unburnt dune crest was sampled at each of the three sites, and in Experiment 2, one unburnt crest was used at each of sites 1 and 2.

#### Foraging station set-up

**Figure 1:**
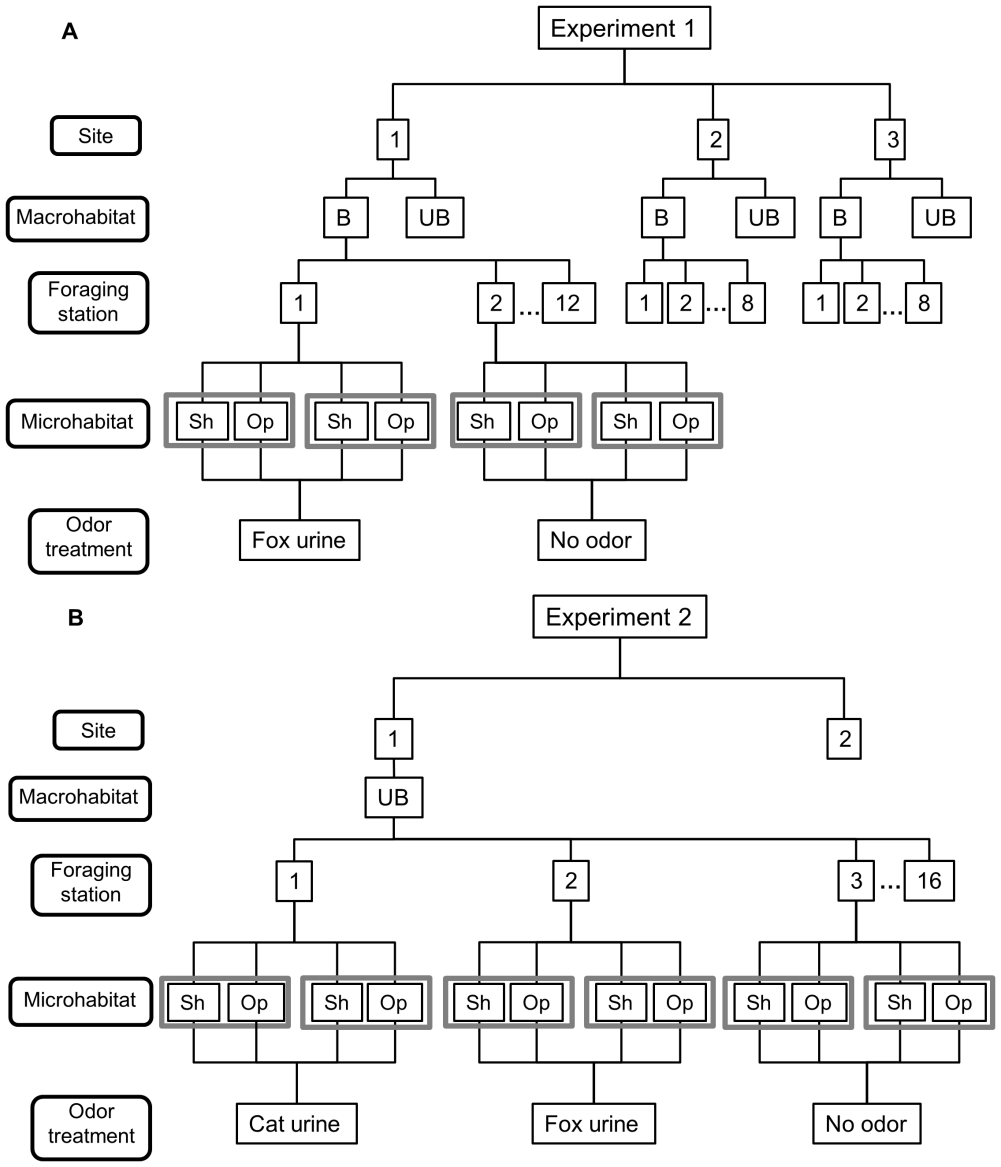
Schematic diagram of the experimental design for (A) Experiment 1 and (B) Experiment 2. Grey boxes indicate pairs of foraging patches set in sheltered microhabitats (Sh) and open microhabitats (Op). The numbers of foraging stations are consistent across unburnt (UB) and burnt (B; if relevant) macrohabitats for individual sites, and the microhabitat design is consistent across each foraging station for all sites. The predator odor treatment is repeated, as shown below, at each foraging station.

For Experiment 1, we established 12 foraging stations on both the burnt and unburnt dune crest at site 1, and 8 stations on the burnt and 8 on the unburnt crests at sites 2 and 3 (total of 56 foraging stations; Fig. 1a). For Experiment 2, we established 16 foraging stations on the dunes at sites 1 and 2 (total of 32 foraging stations; Fig. 1b). In both experiments, a foraging station comprised 2 pairs of artificial food patches. One food patch comprised a single bowl containing 20 unsalted peanut quarters mixed with sand (see [36] for extended methods).

To test for the effects of different levels of microhabitat cover on rodent foraging activity and movements, within each pair of bowls at a foraging station, 1 bowl was set in open sand >1 m from vegetation, and 1 under the cover of shrubs or spinifex hummocks that were >30 cm high with a horizontal spread >1 m [36]. Foraging station pairs were spaced ∼20 m apart, but within a pair the patches were separated by <10 m to allow rodents to choose between open and sheltered microhabitats. Foraging stations were set 100–200 m apart along each dune crest to reduce visits by the same individual rodents to more than one station; *N. alexis* usually ranges <100 m a night [37].

#### Predator odor application

To test the effects of predator odor, we placed a wooden dowel (23 cm long×0.6 cm diameter) upright in sand 2 cm from each bowl. The top of the experimental dowels was soaked overnight in predator urine and control dowels were left untreated [2]. We did not use a pungency control, as unfamiliar odors can have complex effects on prey behavior [38]. To ensure that predators were active across all sites we constructed predator activity plots at each site and recorded cat and fox activity over the duration of both experiments (for results see Table S1 and [49] and for detailed methods on sandplot design see [60]).

In Experiment 1, we tested the responses of *N. alexis* to fox urine, which we syringed directly from the bladders of foxes killed for pest control and had stored at ∼1°C until use. We applied predator odors to alternate stations, such that each station with four bowls represented a single odor treatment (i.e. fox urine or no odor; Fig. 1a). Experiment 2 tested responses to both fox urine and cat urine obtained from euthanized cats collected from the Royal Society for the Prevention of Cruelty to Animals (RSPCA). We applied fox, cat or no odor to alternating stations at sites 1 and 2, such that each station represented one odor treatment (Fig. 1b). We ran Experiment 1 for 3 successive nights with predator odor treatments applied after pre-baiting for 3 nights. In Experiment 2, pre-baiting was run for 7 successive nights and odor was then applied for 5 further nights. Each morning, we recorded the identity of rodents that had visited the bowls from their footprints, each bowl was sieved, and remaining peanut quarters counted to acquire measures of foraging activity (see below). If we identified tracks of *N. alexis*, they were followed and scored to record movement paths (see below).

#### Foraging activity

For this measure we scored the number of bowls visited under each treatment, as gauged by the presence of rodent footprints, and also the giving-up density (GUD), a measure of the trade-off between food acquisition by foragers and the risk of predation they incur by staying at the patch. Here, GUDs were measured as the number of peanut quarters remaining in food bowls at the end of each night of foraging [39].

#### Movement paths

To determine rodent movements in response to predator odor and habitat, we followed tracks of *N. alexis* moving from the food patches [40]. We ascertained the habitat components used by rodents after leaving the food patches by scoring percentage estimates of various components along their movement paths. The components were spinifex, open sand, dead wood, leaf litter, shrubs and herbs, and were scored visually in a 50 cm radius every 1 m along the tracks for up to 7–8 m from the food patch. To assess whether any components were used more or less than available in the environment, we measured a straight line in a randomly selected compass direction of equal length to the observed track from the starting point of the observed track, and then scored the percentage cover of each component using the same method as for the actual rodent tracks.

### Statistical Analyses

We used R (version 2.15.2) for all statistical analyses [41]. Values of *P*≤0.05 were accepted as significant in all analyses.

#### Foraging activity

Presence/absence scores and GUDs of *N. alexis* at each food bowl were pooled over temporal replicates and sites for each experiment, producing four data sets. If predator prints or disturbance were found at any of the foraging stations during the test period or prebaiting stage of the experiment, these data points were removed to avoid confounding the effects of experimental predator odors on rodent responses with those odors present naturally in the system. For the GUD analyses, we included only data points associated with foraging patches where there was indication of activity by *N. alexis*.

To compare the number of bowls visited by *N. alexis* in relation to habitat and odor, we used generalized mixed-effects log-linear models in R package ‘lme4’ [42]. These models accounted for spatial non-independence; i.e., the same rodents could potentially forage at all 4 bowls at a station [43]. Data were added across days on the premise that, whilst habituation to predator odor may occur over time, investigation rates and hence visitations are likely to remain similar across several days [44]. These models used Poisson distribution with a log-link function, with number of bowls visited across temporal replicates used as the response variable. In Experiment 1, odor, macrohabitat and microhabitat were set as fixed factors, each with 2 levels: fox urine and no urine, burnt and unburnt, and open and sheltered, respectively; foraging station was set as a random factor. In Experiment 2, odor and microhabitat were evaluated as fixed factors, with the levels: fox urine, cat urine and no urine, and open and sheltered, respectively; foraging station again was the random factor. *Z*-values were used to assess the significance of factors and their interactions.

To compare the GUDs of *N. alexis* in relation to habitat and odor, we again used generalized mixed-effects log-linear models in the R package ‘lme4’ [42], and used them to account for both spatial and temporal non-independence; i.e., rodents could forage at all bowls at a station over separate days (data were not combined across temporal replicates). Log-linear models were used, as GUD data had heterogeneous variances (Levene’s test, *P*<0.001) that could not be corrected by transformation. The Poisson distribution with a log-link function was used in each of the models, with GUDs at individual bowls the response variable. For Experiment 1, odor, macrohabitat and microhabitat were fixed factors and for Experiment 2, odor and microhabitat were set as fixed factors. Foraging station was treated as a random effect and significance assessed by *Z*-tests.

#### Movement paths

Separate analyses were performed on data from Experiments 1 and 2. Pearson’s correlations were computed for variables in each of the 2 datasets; however, collinearity was relatively small (*r*<0.7), so all variables were retained in models [43].

We calculated the mean percentage cover of each habitat component along each track made by *N. alexis* and along each randomly selected straight line. To compare differences in the habitat components used by the rodents and those along the randomly selected lines, and to account for temporal and spatial non-independence at foraging stations, we used a generalized linear mixed model [45] in the R package ‘glmmML’ [46]. A binary distribution (rodent track versus random line) was the response variable, and factors were percentage spinifex, percentage open sand, percentage dead wood, percentage leaf litter, percentage shrub, and percentage herb (continuous variables). Odor (categorical) and macrohabitat (categorical) were also added as factors for Experiment 1. Station was the random factor in all models. We selected the best models based on the corrected Akaike’s Information Criterion (AIC_c_) [47] using the R package ‘MuMIn’ [48]. Candidate models were considered if the difference in AIC between the candidate model and the top model (Δ_c_) was <2 [47].

As there was no clear “best” model where Δ_c_<2, we performed model averaging to acknowledge uncertainty [47]. This produces an estimate of the effect size (direction and magnitude) for each predictor variable by averaging the coefficient estimates from all candidate models (in this case, for all models where Δ_c_<2), for each predicator variable, weighted by the w_i_ of each model [47]. Akaike weights (w_i_) represent the ratio of Δ_c_ values for each model relative to the whole set of Δ_c_<2 candidate models. Estimates of uncertainty were based on the unconditional standard errors of the parameter estimates [47]. We determined the relative importance of each predictor variable by summing the Akaike weights (∑w_i_) from all model combinations (Δ_c_<2) where the variable occurred. Variables were ranked according to their ∑w_i_, where the more important variables had larger weights. Model averaging was not applied in Experiment 1 as the models included categorical variables (macrohabitat and odor), which cannot be averaged.

## Results

### Foraging Activity

In Experiment 1 *Notomys alexis* visited foraging stations equally, whether bowls were in different macrohabitats, microhabitats or in the presence of fox odor or not; nor were there any interactions between these factors (Table 1). In Experiment 2, *N. alexis* again showed no preference for stations with or without predator odors and no interactions between odor and microhabitat (Table 1). However, there was a preference for open microhabitats (*Z = *1.77, *P* = 0.048), with *N. alexis* visiting 130 bowls in sheltered microhabitats compared to 169 in the open (Table 1).

**Table 1:** Results from log-linear models comparing visits to patches by *Notomys alexis* in different habitats and with and without predator odors.

	Terms	Estimate	SE	*Z*	*P*
**Experiment 1**	Unburnt habitat	0.08	0.45	0.30	0.768
	Fox odor	–0.30	–0.30	–0.79	0.429
	Open microhabitat	–0.24	–0.24	–0.84	0.398
	Unburnt habitat × Fox odor	0.29	0.65	0.50	0.619
	Unburnt habitat × Open microhabitat	0.31	0.39	0.80	0.424
	Fox odor × Microhabitat	0.59	0.42	1.43	0.153
	Unburnt habitat × Fox odor × Open microhabitat	–0.55	0.56	–1.00	0.319
**Experiment 2**	Fox odor	–0.50	0.31	–1.59	0.112
	Cat odor	–0.30	0.30	–0.99	0.321
	Open microhabitat	0.32	0.18	1.77	0.048
	Fox odor × Open microhabitat	–0.13	0.30	–0.45	0.653
	Cat odor × Open microhabitat	–0.07	0.27	–0.27	0.787

We compiled a total of 175 GUD scores in Experiment 1. Giving-up densities were high and invariant across microhabitats, macrohabitats and odor treatment, and there were no significant interactions between factors (Table 2). The mean GUD across the experiment was 18.6±0.1 SE peanut quarters (out of a total of 20 quarters). For Experiment 2, GUDs (*n* = 279 scores) were invariant across microhabitats and odor treatments; there were also no significant interactions between these factors (Table 2). The mean GUD for Experiment 2 was 18.1±0.1 SE peanut quarters.

**Table 2:** Results from generalized mixed-effects log-linear models comparing giving-up densities for *Notomys alexis* in different habitats and with and without predator odors.

	Terms	Estimate	SE	*Z*	*P*
**Experiment 1**	Unburnt habitat	–0.06	0.06	–1.00	0.316
	Fox odor	–0.01	0.08	–0.10	0.922
	Open microhabitat	–0.05	0.07	–0.75	0.454
	Unburnt habitat × Fox odor	0.04	0.10	0.43	0.670
	Unburnt habitat × Open microhabitat	0.08	0.09	0.90	0.371
	Fox odor × Microhabitat	0.01	0.11	0.13	0.898
	Unburnt habitat × Fox odor × Open microhabitat	–0.04	0.15	–0.25	0.799
**Experiment 2**	Fox odor	0.03	0.05	0.65	0.514
	Cat odor	–0.01	0.05	–0.15	0.881
	Open microhabitat	0.03	0.04	0.56	0.574
	Fox odor × Open microhabitat	–0.01	0.07	–0.15	0.880
	Cat odor × Open microhabitat	–0.01	0.07	–0.21	0.830

### Movement Paths

In Experiment 1, 152 individual tracks of *N. alexis* were followed over about 1,100 m. There were 256 models comparing *N. alexis* tracks with random transects. Of these, 15 had a Δ_c_<2 (Table 3). *Notomys alexis* selected more open sand than was available and also more spinifex and less shrub than was available. Macrohabitat was considered in 5 of the top models and indicated that *N. alexis* used more open sand, spinifex and shrub in the burnt habitats compared to the unburnt habitats. Odor was included in only 1 model and the SE was greater than the estimate (Table 3).

**Table 3:** The 15 best (Δ_c_<2) generalized mixed models comparing habitat components traversed by *Notomys alexis* with those available on random transects.

Modelno.	Intercept	Burnt habitat	Herbs	Leaf litter	Fox odor	Open Sand	Shrub	Spinifex	Dead wood	AIC_c_	Δ_c_	*w_i_*
1	–0.03±0.12					0.81±0.17		0.23±0.15		398.81	0.00	0.04
2	–0.03±0.12					0.56±0.15	–0.24±0.16			398.82	0.01	0.04
3	–0.02±0.12					0.67±0.14				399.18	0.37	0.03
4	–0.19±0.18	0.29±0.25				0.84±0.17		0.22±0.15		399.55	0.74	0.03
5	–0.04±0.12		–0.18±0.16			0.56±0.15	–0.27±0.16			399.57	0.77	0.03
6	–0.20±0.18	0.31±0.25				0.72±0.17				399.66	0.85	0.03
7	–0.18±0.18	0.27±0.25				0.61±0.16	–0.22±0.16			399.76	0.95	0.03
8	–0.04±0.12			0.15±0.14		0.90±0.20		0.28±0.15		399.81	1.00	0.03
9	–0.03±0.12		–0.15±0.16			0.68±0.14				400.31	1.50	0.02
10	–0.03±0.12					0.69±0.24	–0.15±0.21	0.14±0.20		400.38	1.57	0.02
11	–0.04±0.12		–0.11±0.16			0.80±0.17		0.21±0.15		400.43	1.62	0.02
12	–0.03±0.12					0.53±0.16	–0.27±0.16		–0.10±0.18	400.58	1.78	0.02
13	–0.18±0.18	0.26±0.25	–0.17±0.16			0.61±0.16	–0.25±0.16			400.61	1.80	0.02
14	0.02±0.19				–0.09±0.25	0.56±0.15	–0.25±0.16			400.75	1.95	0.02
15	–0.18±0.19	0.26±0.25		0.13±0.18		0.92±0.36		0.27±0.27		400.79	1.98	0.02

Table includes coefficients and standard errors, with AIC_c_ values, change in AIC_c_ values (Δ_c_) and Akaike weight (*w_i_*), for Experiment 1.

During Experiment 2, we followed 319 tracks of *N. alexis* over 2,200 m. In total, 128 models were generated comparing *N. alexis* tracks with random transects. Of these, 5 models had a Δ_c_<2. Open sand, shrub, spinifex, and to a lesser extent leaf litter, dead wood and herbs were important variables contributing to the models. Odor was not included in any models (Table 4). *Notomys alexis* used more open sand and less spinifex and shrub than was available in the environment (Table 4).

**Table 4:** The mean parameter estimates and standard errors for key explanatory variables and ranking of predictor variables explaining tracks made by *Notomys alexis*.

	Estimate	SE	*Z*	*P*	Ranking
**Open Sand**	1.52	0.49	3.11	0.002	1.00
**Shrub**	−1.08	0.56	1.93	0.054	1.00
**Spinifex**	−1.09	0.49	2.24	0.025	0.83
**Leaf litter**	−0.58	0.46	1.26	0.207	0.33
**Dead wood**	−0.13	0.25	0.54	0.592	0.31
**Herbs**	−0.10	0.25	0.41	0.679	0.15
**Intercept**	1.35	0.51	2.68	0.007	

Ranks are provided according to the sum of Akaike weights (Σwi) for each variable. Estimates and standard errors are derived from all combinations of the regression models for *Notomys alexis* in Experiment 2 (number of models = 5).

## Discussion

Our results indicate that predator odor had no influence on the foraging behavior, movement paths or habitat use of *Notomys alexis*, and thus provide no support for our general hypothesis that predator odor would provide a cue to gauge predation risk. These results may reflect the ephemeral nature of predation in our desert system or weak or limited selection on rodents for the use of predator odor cues. However, there was some evidence to suggest preferential use of open habitats by *N. alexis*. After first discussing the lack of support for the predator-odor hypothesis, we use these observations to propose that this rodent uses alternative strategies, linked to morphological traits, that rely on escape after encounter with a predator rather than avoidance in the first instance.

### Predator Odor as a Cue for Increased Predation Risk

If odor cannot be readily detected in a system, it is unlikely to be used as a cue for predation risk. The hot dry air in deserts may render communication by odor of short-term importance only. In particular, odors derived from urine probably decay at a faster rate than fecal odors due to evaporation. However, rodents have been shown to respond to urine-based odor treatments in similar experiments investigating predator odor cues in arid environments elsewhere [2], and in the present study we applied extra urine at dusk each night to ensure that it was fresh.

Another possibility is that prey responses to predator odor may not be elicited if the scents represent predators that are not current, substantial threats to the prey. However, analysis of both cat and fox feces at the study sites indicated that *N. alexis* occurred frequently in the diet of both predators [49]. Further, predator activity at the time of study was relatively high [34], suggesting that foxes and cats probably represented a substantial and current threat to prey species. However, without directly measuring the number and life-history stages of prey animals depredated by foxes and cats, the actual pressure that the predators are imposing cannot be ascertained. For example, if predators primarily hunt young or post-reproductive animals that would have died soon in any case (the ‘doomed surplus’), predation-impacts would not be additive and selective pressure on prey to respond to odor cues from their predators would be weak [50].

If *N. alexis* is incapable of detecting differences in cat and fox scents, or an absence of their odors, this may explain why they did not respond to the experimentally applied predator odors. Indeed, several studies have shown that Australian rodents often do not appear to respond to fox or cat scents [38], [51], perhaps because of the lack of a shared evolutionary history between the rodents and the newly invasive predators. However, laboratory and field-based experiments demonstrate that rodents can discriminate between predator scents, including those from cat [52] and fox [53]. There are also specific “fear-inducing” chemicals in the feces of foxes that may be recognized by animals that are evolutionarily naive to predation by foxes [54]. As many mammals respond to the odor of both novel and native predators through detection of common chemical constituents [55], [56], it therefore seems likely that the desert rodents studied here could detect the odors of fox and cat that we presented to them.

Before the introduction and spread of the red fox and feral cat into arid Australia, native rodents would have encountered relatively few mammalian predators that regularly deposited scent. The dingo (*Canis dingo*) would have been present and also quolls (*Dasyurus* spp.) to a lesser degree; however, both are unlikely to have exerted much predation pressure on *N. alexis*. The western quoll (*Dasyurus geoffroii*), the only quoll present in arid regions, probably had a diet comprised primarily of invertebrates, as it does now in its remnant range [57]. Dietary analysis of the dingo at the present study site indicates a preference for larger prey animals [49]. Thus, the lack of response to cat and fox odor by rodent species might not reflect a failure to specifically recognize novel predator odor, but an absence of important scent-producing predators in the evolutionary history of the rodent species. This might, in association with other factors, explain why introduced predators have such great impacts on prey in Australian arid environments [58] in comparison with those in semi-arid and forest environments where prey animals have probably been exposed to stronger selection from a greater range of mammalian predators.

If selection is strong, anti-predator behaviors might evolve rapidly and prey could respond to novel predator odors regardless of the historic presence of these predators [12]. Indeed, in the Simpson Desert, high per capita predation risk and strong selection are likely, as predators are drawn by “booming” prey populations following large rains [19]. However, during drought periods, when prey densities plummet and free water becomes scarce, foxes and cats in the Simpson Desert likely increase their mobility [59] and localized predator densities then can fall to zero [60]. It is probable that prey experience several years during droughts with little or no exposure to predators [60]. Therefore, the lack of response to predator odor that we observed may reflect ephemeral predation pressure that is imposed on prey species for periods that are not sufficiently sustained for predation to have a consistent selective effect.

It is also possible, however, that prey can detect predator odors, but the usefulness of the cue might be outweighed by opportunities that could be missed due to responding to the cue [39]. If the costs of avoiding odor cues outweigh the risks of encountering a predator, this might encourage the decision to chance a contact with the predator [39]. Resources decline during droughts, so areas that might once have been considered too risky to exploit then appear favorable. When we exposed *N. alexis* to predator odor, we also provided them with a rich food patch. As they were not given the choice of foraging at stations with and without odor (each independent station was treated with either odor or no odor), animals may have chosen to continue foraging because the benefits of feeding outweighed those of avoiding the odor. To properly test this hypothesis, it would be necessary to determine the food resources available to rodents and thus whether resource limitation is a factor.

The final and perhaps most compelling explanation for our results is that avoidance of predator odor may be unnecessary as habitat variability allows prey animals to detect and escape from predators even if they are present and encountered. Most studies indicate that prey responses to habitat structure are typically more prominent than responses to predator odor cues, suggesting that actual predator presence often may be irrelevant in terms of prey foraging decisions and habitat use [23], [61]. Many rodent species show preferences for certain microhabitats that decrease the chance that they will be detected or captured by predators (e.g. [5], [23], [62]). This leads to the most positive findings of our study, that *N. alexis* showed preferences for certain components of the habitat.

### Habitat Components and the Management of Predation Risk

Foxes, dingoes and cats occur in the study area, as do predatory marsupials (e.g. mulgara *Dasycercus blythi*) and avian predators such as the barn owl (*Tyto alba*) [63], [64]. In general, encounters with these visually oriented predators are more likely to occur in open habitats than under or in the protective cover of spinifex and shrubs [65], [66]. However, we observed a clear preference for open habitats by *N. alexis*. This result was similar to that of a previous study, which found that *N. alexis* typically approached open rather than sheltered food patches [29].

*Notomys alexis* probably uses open habitats for two reasons. First, use of open space may increase the probability that the animal rapidly detects its predators. While encounters between cats and foxes and their prey might occur more frequently in open habitats, cats in particular can use plant cover to stealthily approach their prey (e.g. [67]). Therefore, while shelter might provide rodents with refuge, it may also hinder predator detection [68]. Further, *N. alexis* has well developed hearing and keen visual acuity, which would support early detection of predators in open habitats [47]. Second, *N. alexis* may have greater success in escaping from predators in open compared to sheltered habitats. *Notomys alexis* has morphological traits such as powerful hind limbs that enable bipedal movement and allow rapid escape following detection of a predator [69]. This mode of locomotion is probably most effective in open habitats that permit higher running speeds [70]. Studies of habitat use under the risk of predation have focused almost exclusively on habitat-specific attack rates by predators and predator abundance [71], whereas habitat-specific escape by prey has rarely been considered. However, there is evidence that some rodents prefer to use more open environments, and sheltered environments have been linked to a reduced likelihood of escape from predators [70], [72], [73].

It is also possible that bipedal *N. alexis* makes greater use of open space to gain competitive advantage over other animals, such as the slower moving quadrupedal rodents that are present in the desert system. Greater mobility and speed may allow *N. alexis* to access resource patches that are too risky for slower moving animals to exploit [74]. In particular, as food availability declines, such as during drought, risky habitats may contain patches of unexploited food resources and thus present an acceptable tradeoff between predation risk and food income for bipedal rodents. To determine whether any competitive advantage is gained by the use of more open space by *N. alexis*, food availability in open and sheltered habitats should be surveyed and compared.

Although *N. alexis* showed some preferences for different habitat components, it returned high, non-significantly different GUDs in open and sheltered microhabitats. This result also contradicts prior studies, which indicate that sheltered microhabitats are preferred for foraging by *N. alexis* [29]. GUD is a measure of the risk perceived by an animal *as it feeds* at a food patch. Therefore, whilst *N. alexis* favors movement in open space, it may have different preferences for feeding. Feeding is likely to be riskier than traveling between food patches due to the tradeoff between feeding and vigilant behavior. Thus, a possible explanation for the lack of disparity between GUDs at open and sheltered sites is that the risk perceived by rodents whilst feeding was similarly high in both microhabitats. Prey species on the dune crest, where most predator activity is typically focused [60], may experience a uniformly high level of risk whilst feeding in the open and under shelter. Prior studies sampled entire dune habitats and did not consider the use of microhabitats on dune crests [29]. Because vegetation on the dune crests is so sparse [75], detection by predators may be similar in both open and sheltered microhabitats and prey may not perceive large differences in risk across these microhabitats. If this is so, animals might perceive both microhabitats as similarly risky and forage opportunistically as they encounter food bowls, spending as little time as possible at them to minimize detection by predators. This suggestion also explains the uniformly high GUDs and may also account for why there were no differences in the responses of rodents in the burnt and unburnt crest habitats.

As next steps, we recommend that exploration of rodent responses to predator odor and habitat cues is needed during different stages of rodents’ population boom and bust cycles to uncover possible shifts in response under conditions of varying predation risk.

## Supporting Information

Table S1 **Cat and fox activity recorded at study sites during Experiments 1 and 2.** Activity measure was provided by binary counts of foot prints on randomly located sandplot grids, with 6 sandplot transects per grid. Sandplots were checked for presence/absence of fox and cat footprints on 3–10 consecutive mornings. Data were averaged over the 3–10 day sampling period, for each grid, providing an activity index (see [60] for further details on sandplot methods).
(DOCX)

## References

[1] Apfelbach R, Blanchard CD, Blanchard RJ, Hayes RA, McGregor IS (2005) The effects of predator odors in mammalian prey species: a review of field and laboratory studies. Neuroscience & Biobehavioral Reviews 29: 1123–1144. 16085312 10.1016/j.neubiorev.2005.05.005

[2] Herman CS, Valone TJ (2000) The effect of mammalian predator scent on the foraging behavior of *Dipodomys merriami* . Oikos 91: 139–145.

[3] Stoddart DM (1982) Does trap odour influence estimation of population size of the short-tailed vole, *Microtus agrestis*? Journal of Animal Ecology 51: 375–386.

[4] Dickman CR, Doncaster CP (1984) Responses of small mammals to red fox (*Vulpes vulpes*) odour. Journal of Zoology 204: 521–531.

[5] Jones M, Dayan T (2000) Foraging behavior and microhabitat use by spiny mice, *Acomys cahirinus* and *A. russatus*, in the presence of Blanford’s fox (*Vulpes cana*) odor. Journal of Chemical Ecology 26: 455–469.

[6] Letnic M, Crowther MS, Dickman CR, Ritchie EG (2011) Demonising the dingo: how much wild dogma is enough? Current Zoology 57: 668–670.

[7] Dickman CR (1996) Overview of the impacts of feral cats on Australian native fauna. Australian Nature Conservation Agency, Canberra.

[8] Dickman CR (1996) Impact of exotic generalist predators on the native fauna of Australia. Wildlife Biology 2: 185–195.

[9] Salo P, Korpimäki E, Banks PB, Nordström M, Dickman CR (2007) Alien predators are more dangerous than native predators to prey populations. Proceedings of the Royal Society B: Biological Sciences 274: 1237–1243. 17360286 10.1098/rspb.2006.0444 PMC1950296

[10] Nersesian CL, Banks PB, McArthur C (2012) Behavioural responses to indirect and direct predator cues by a mammalian herbivore, the common brushtail possum. Behavioral Ecology and Sociobiology 66: 47–55.

[11] Woolhouse AD, Morgan DR (1995) An evaluation of repellents to suppress browsing by possums. Journal of Chemical Ecology 21: 1571–1583. 24233684 10.1007/BF02035153

[12] Anson JR, Dickman CR (2013) Behavioral responses of native prey to disparate predators: naiveté and predator recognition. Oecologia 171: 367–377. 22865005 10.1007/s00442-012-2424-7

[13] Dickman CR (1993) Raiders of the last ark: cats in island Australia. Australian Natural History 24: 44–52.

[14] Blumstein DT, Mari M, Daniel JC, Ardron JG, Griffin AS, et al (2002) Olfactory predator recognition: wallabies may have to learn to be wary. Animal Conservation 5: 87–93.

[15] Mella VSA, Cooper CE, Davies SJJF (2011) Predator odour does not influence trappability of southern brown bandicoots (*Isoodon obesulus*) and common brushtail possums (*Trichosurus vulpecula*). Australian Journal of Zoology 58: 267–272.

[16] Polis G (1993) Scorpions as model vehicles to advance theories of population and community ecology: the role of scorpions in desert communities. Memoirs of the Queensland Museum 33: 401–410.

[17] Cloudsley-Thompson JL (1976) Deserts and grasslands. Part 1: desert life. Doubleday, New York.

[18] Ayal Y (2007) Trophic structure and the role of predation in shaping hot desert communities. Journal of Arid Environments 68: 171–187.

[19] Letnic M, Dickman CR (2006) Boom means bust: interactions between the El Niño/Southern Oscillation (ENSO), rainfall and the processes threatening mammal species in arid Australia. Biodiversity & Conservation 15: 3847–3880.

[20] Letnic M, Story P, Story GL, Field J, Brown O, et al (2011) Resource pulses, switching trophic control, and the dynamics of small mammal assemblages in arid Australia. Journal of Mammalogy 92: 1210–1222.

[21] Sala OE, Aguiar MR (1996). Origin, maintenance, and ecosystem effect of vegetation patches in arid lands; 1996. In: West N (ed) Rangelands in a sustainable biosphere. Society for Range Management, Denver, 29–32.

[22] Fey K, Banks PB, Korpimäki E (2006) Different microhabitat preferences of field and bank voles under manipulated predation risk from an alien predator. Annales Zoologici Fennici 43: 9–16.

[23] Orrock JL, Danielson BJ, Brinkerhoff RJ (2004) Rodent foraging is affected by indirect, but not by direct, cues of predation risk. Behavioral Ecology 15: 433–437.

[24] Greenville AC, Dickman CR, Wardle GM, Letnic M (2009) The fire history of an arid grassland: the influence of antecedent rainfall and ENSO. International Journal of Wildland Fire 18: 631–639.

[25] Pastro LA, Dickman CR, Letnic M (2013) Effects of wildfire, rainfall and region on desert lizard assemblages: the importance of multi-scale processes. Oecologia 173: 603–614. 23494288 10.1007/s00442-013-2642-7

[26] Pastro LA, Dickman CR, Letnic M (2011) Burning for biodiversity or burning biodiversity? Prescribed burn vs. wildfire impacts on plants, lizards, and mammals. Ecological Applications 21: 3238–3253.

[27] Cupples JB, Crowther MS, Story GL, Letnic M (2011) Dietary overlap and prey selectivity among sympatric carnivores: could dingoes suppress foxes through competition for prey? Journal of Mammalogy 92: 590–600.

[28] Pavey CR, Eldridge SR, Heywood M (2008) Population dynamics and prey selection of native and introduced predators during a rodent outbreak in arid Australia. Journal of Mammalogy 89: 674–683.

[29] Dickman CR, Greenville AC, Beh C-L, Tamayo B, Wardle GM (2010) Social organization and movements of desert rodents during population “booms” and “busts” in central Australia. Journal of Mammalogy 91: 798–810.

[30] Van Dyck S, Strahan R (2008) The Mammals of Australia., 3rd edn. Reed New Holland, Sydney.

[31] Murray BR, Dickman CR (1994) Food preferences and seed selection in two species of Australian desert rodents. Wildlife Research 21: 647–655.

[32] Purdie RW (1984) Land systems of the Simpson Desert region. Institute of Biological Resources.

[33] Dickman CR, Downey FJ, Predavec M (1993) The hairy-footed dunnart, *Sminthopsis hirtipes* (Marsupialia: Dasyuridae) in Queensland. Australian Mammalogy 16: 69–72.

[34] Dickman CR, Wardle GM, Foulkes JN, de Preu ND (2014) Desert complex environments. In: Lindenmayer DB, Burns E, Thurgate N, Lowe AN, (editors). Biodiversity and environmental change: monitoring, challenges and direction. CSIRO Publishing, Melbourne, 389–448.

[35] Kotler BP, Brown J, Mukherjee S, Berger-Tal O, Bouskila A (2010) Moonlight avoidance in gerbils reveals a sophisticated interplay among time allocation, vigilance and state-dependent foraging. Proceedings of the Royal Society B: Biological Sciences 277: 1469–1474. 20053649 10.1098/rspb.2009.2036 PMC2871830

[36] Kotler BP, Dickman CR, Brown JS (1998) The effects of water on patch use by two Simpson Desert granivores (*Corvus coronoides* and *Pseudomys hermannsburgensis*). Australian Journal of Ecology 23: 574–578.

[37] Letnic M (2001) Long distance movements and the use of fire mosaics by small mammals in the Simpson Desert, central Australia. Australian Mammalogy 23: 125–134.

[38] Banks PB (1998) Responses of Australian bush rats, *Rattus fuscipes*, to the odor of introduced *Vulpes vulpes* . Journal of Mammalogy 79: 1260–1264.

[39] Brown JS (1988) Patch use as an indicator of habitat preference, predation risk, and competition. Behavioral Ecology and Sociobiology 22: 37–47.

[40] Triggs B (1996) Tracks, scats and other traces: a field guide to Australian mammals. Oxford University Press, Melbourne.

[41] R Development CoreTeam (2012) R: a language and environment for statistical computing. http://www.R-project.org/. Vienna, Austria: R Foundation for Statistical Computing.

[42] Bytheway JP, Carthey AJR, Banks PB (2013) Risk vs. reward: how predators and prey respond to aging olfactory cues. Behavioral Ecology and Sociobiology 67: 715–725.

[43] Bates D, Maechler M, Bolker B (2012) lme4: Linear mixed-effects models using S4 classes. R package version 0.999999-0.

[44] Quinn GP, Keough MJ (2002) Experimental design and data analysis for biologists. Cambridge University Press, Cambridge.

[45] McCulloch CE, Searle SR (2001) Generalized, linear and mixed models. John Wiley & Sons, New York.

[46] Broström G (2008) glmmML: Generalized linear models with clustering. R package version 0.81-2.

[47] Burnham KP, Anderson DR (2002) Model selection and multimodel inference: a practical information-theoretic approach. Springer-Verlag, New York.

[48] Barton K (2009) MuMIn: multi-model inference. R package version 0.12.0.

[49] Spencer EE (2013) Interactions between mammalian predators and prey in the Simpson Desert in central Australia. Honours thesis, Faculty of Science, University of Sydney.

[50] Banks PB (1999) Predation by introduced foxes on native bush rats in Australia: do foxes take the doomed surplus? Journal of Applied Ecology 36: 1063–1071.

[51] McEvoy J, Sinn DL, Wapstra E (2008) Know thy enemy: behavioural response of a native mammal (*Rattus lutreolus velutinus*) to predators of different coexistence histories. Austral Ecology 33: 922–931.

[52] Dielenberg RA, McGregor IS (2001) Defensive behavior in rats towards predatory odors: a review. Neuroscience & Biobehavioral Reviews 25: 597–609. 11801285 10.1016/s0149-7634(01)00044-6

[53] Dickman CR (1992) Predation and habitat shift in the house mouse, *Mus domesticus* . Ecology 73: 313–322.

[54] Fendt M, Siegl S, Steiniger-Brach B (2005) Noradrenaline transmission within the ventral bed nucleus of the stria terminalis is critical for fear behavior induced by trimethylthiazoline, a component of fox odor. The Journal of Neuroscience 25: 5998–6004. 15976089 10.1523/JNEUROSCI.1028-05.2005 PMC6724787

[55] Russell BG, Banks PB (2007) Do Australian small mammals respond to native and introduced predator odours? Austral Ecology 32: 277–286.

[56] Hayes RA, Nahrung HF, Wilson JC (2006) The response of native Australian rodents to predator odours varies seasonally: a by-product of life history variation? Animal Behaviour 71: 1307–1314.

[57] Hancock C (1991) A report on the distribution, seasonal occurrence and diet of the Chuditch (*Dasyurus geoffroii*). Student report, Murdoch University, Perth.

[58] McKenzie NL, Burbidge AA, Baynes A, Brereton RN, Dickman CR, et al (2007) Analysis of factors implicated in the recent decline of Australia’s mammal fauna. Journal of Biogeography 34: 597–611.

[59] Edwards GP, De Preu N, Shakeshaft BJ, Crealy IV, Paltridge RM (2008) Home range and movements of male feral cats (*Felis catus*) in a semiarid woodland environment in central Australia. Austral Ecology 26: 93–101.

[60] Mahon PS (1999) Predation by feral cats and red foxes, and the dynamics of small mammal populations in arid Australia: Ph.D. thesis, University of Sydney, Sydney.

[61] Verdolin JL (2006) Meta-analysis of foraging and predation risk trade-offs in terrestrial systems. Behavioral Ecology and Sociobiology 60: 457–464.

[62] Thorson JM, Morgan RA, Brown JS, Norman JE (1998) Direct and indirect cues of predatory risk and patch use by fox squirrels and thirteen-lined ground squirrels. Behavioral Ecology 9: 151–157.

[63] Woolley PA (2005) The species of *Dasycercus* Peters, 1875 (Marsupialia: Dasyuridae). Memoirs of Museum Victoria 62: 213–221.

[64] Woolley PA (2006) Studies on the crest-tailed mulgara *Dasycercus cristicauda* and the brush-tailed mulgara *Dasycercus blythi* (Marsupialia: Dasyuridae). Australian Mammalogy 28: 117–120.

[65] Dickman CR (2009) House cats as predators in the Australian environment: impacts and management. Human-Wildlife Conflicts 3: 41–48.

[66] Dickman CR, Predavec M, Lynam AJ (1991) Differential predation of size and sex classes of mice by the barn owl, *Tyto alba* . Oikos 62: 67–76.

[67] Branch LC (1995) Observations of predation by pumas and Geoffroy’s cats on the plains vizcacha in semi-arid scrub of central Argentina. Mammalia 59: 152–155.

[68] Embar K, Kotler BP, Mukherjee S (2011) Risk management in optimal foragers: the effect of sightlines and predator type on patch use, time allocation, and vigilance in gerbils. Oikos 120: 1657–1666.

[69] Stanley M (1971) An ethogram of the hopping mouse, *Notomys alexis* . Zeitschrift für Tierpsychologie 29: 225–258. 5168510 10.1111/j.1439-0310.1971.tb01735.x

[70] Schooley RL, Sharpe PB, Horne BV (1996) Can shrub cover increase predation risk for a desert rodent? Canadian Journal of Zoology 74: 157–163.

[71] Lima SL, Dill LM (1990) Behavioral decisions made under the risk of predation: a review and prospectus. Canadian Journal of Zoology 68: 619–640.

[72] Taraborelli P, Corbalán V, Giannoni S (2003) Locomotion and escape modes in rodents of the Monte Desert (Argentina). Ethology 109: 475–485.

[73] Djawdan M (1993) Locomotor performance of bipedal and quadrupedal heteromyid rodents. Functional Ecology: 195–202.

[74] Kotler BP (1984) Risk of predation and the structure of desert rodent communities. Ecology 65: 689–701.

[75] Buckley R (1981) Soils and vegetation of central Australian sandridges III. Sandridge vegetation of the Simpson Desert. Australian Journal of Ecology 6: 405–422.

