# Supplementary material for: Risky Business: Do Native Rodents Use Habitat and Odor Cues to Manage Predation Risk in Australian Deserts?
Source: PLoS One. 2014 Feb 28;9(2):e90566. doi: 10.1371/journal.pone.0090566 (PMC3938783; doi:10.1371/journal.pone.0090566)
Supplement: Table S1 — Cat and fox activity recorded at study sites during Experiments 1 and 2. Activity measure was provided by binary counts of foot prints on randomly located sandplot grids, with 6 sandplot transects per grid. Sandplots were checked for presence/absence of fox and cat footprints on 3–10 consecutive mornings. Data were averaged over the 3–10 day sampling period, for each grid, providing an activity index (see [60] for further details on sandplot methods). (DOCX) [file pone.0090566.s001.docx]

**Table S1.**

| **Experiment** | **Location** | **Grid** | **Fox activity** | **Cat activity** |
| --- | --- | --- | --- | --- |
| 1 | Site 1 | 1 | 0.3 | 0.7 |
|  |  | 2 | 1.7 | 2.0 |
|  |  | 3 | 0.7 | 1.0 |
|  |  | 4 | 0.3 | 2.0 |
|  | Site 3 | 1 | 0.3 | 0.7 |
|  |  | 2 | 0.3 | 1.0 |
| 2 | Site 1 | 1 | 0.3 | 1.0 |
|  |  | 2 | 0.0 | 1.0 |
|  |  | 3 | 0.3 | 0.7 |
|  | Site 2 | 1 | 0.2 | 0.8 |
